# Additions to the fauna and biology of stoneflies (Plecoptera) in Taizi River Basin, Liaoning, with seven new species records to China

**DOI:** 10.3897/BDJ.10.e95120

**Published:** 2022-11-11

**Authors:** Qing-Bo Huo, Abdur Rehman, Meng-Yuan Zhao, Yu-Ben Yang, Ya-Nan Xiang, Yu-Zhou Du, Jian-Feng Wang, Dávid Murányi, Valentina A. Teslenko

**Affiliations:** 1 School of Horticulture and Plant Protection & Institute of Applied Entomology, Yangzhou University, Yangzhou 225009, China School of Horticulture and Plant Protection & Institute of Applied Entomology, Yangzhou University Yangzhou 225009 China; 2 Joint International Research Laboratory of Agriculture and Agri-Product Safety, the Ministry of Education, Yangzhou University, Yangzhou 225009, China Joint International Research Laboratory of Agriculture and Agri-Product Safety, the Ministry of Education, Yangzhou University Yangzhou 225009 China; 3 Shenyang University, Liaoning Key Laboratory of Urban Integrated Pest Management and Ecological Security, Liaoning, Shenyang110044, Liaoning, China Shenyang University, Liaoning Key Laboratory of Urban Integrated Pest Management and Ecological Security, Liaoning Shenyang110044, Liaoning China; 4 Department of Zoology, Eszterházy Károly Catholic University, Leányka u. 6, H-3300, Eger, Hungary Department of Zoology, Eszterházy Károly Catholic University, Leányka u. 6, H-3300 Eger Hungary; 5 Institute of Biology and Soil Science, Far Eastern Branch, Russian Academy of Sciences, Vladivostok, Russia Institute of Biology and Soil Science, Far Eastern Branch, Russian Academy of Sciences Vladivostok Russia

**Keywords:** Plecoptera, Liaoning, Leuctridae, Nemourinae, Chloroperlidae, Perlidae, Perlodidae, northeast China

## Abstract

**Background:**

An investigation report of stonefly fauna in Benxi Manchu Autonomous County, Liaoning Province, northeast China (used to be called Manchuria, now includes Liaoning, Jilin, Heilongjiang Provinces and parts of Inner Mongolia, which are adjacent to the Russian Far East and the Korean Peninsula). Materials were studied with field observation in 2018 and 2019.

**New information:**

This paper records five families, nine genera and 14 species of stoneflies from Taizi River, Liaoning Province. Nine species have been recorded for the first time in China and the biology of several common species is also reported for the first time.

## Introduction

Taizi River is located in northeast China and is the largest freshwater river in Liaoning Province. Its source is located in the Benxi Manchu Autonomous County to Huanren County in the east of Liaoning, belonging to the Changbai Mountains. There are multiple hills and branches of streams on both sides of Taizi River, with mixed broadleaf-conifer forest (Wu and Tian 2017), of which water quality is suitable for the habitat of aquatic insects, especially stoneflies (Yu et al. 2019). However, the sampling rate of stonefly fauna in Liaoning Province has been low for a long time and few species have been recorded before (Wu 1938, Wu 1973, Du 1999, Yang and Li 2018).

Zheng (2011) has collected only two stonefly genera (*Oyamia* sp., *Suwallia* sp.) from the southern tributary of Taizi River, but found no stonefly in its northern part. Yu et al. (2019) have further recorded more EPT species in the southern Taizi River (Yanghugou Village) and provided part COI sequences, including four families and four genera of Plecoptera, including Nemouridae (*Amphinemura*), Chloroperlidae (*Alloperla*), Perlidae (*Kamimuria*) and Perlodidae (*Stavsolus*), but unfortunately, no species have been identified.

**Figure 1.**
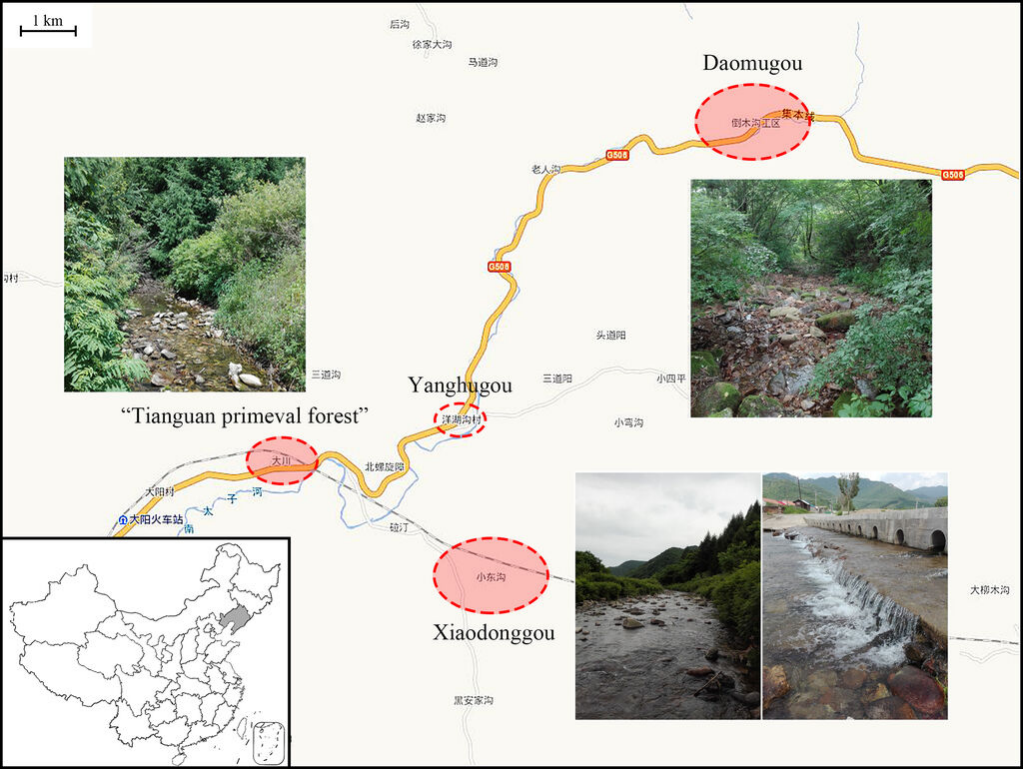
The map of the sampling localities in Taizi River (map provided by www.tianditu.gov.cn).

From 2018 to 2019, more localities of Taizi River Basin including Xiaodonggou (near Yanghugou Village), Tianguan Virgin Forest and Daomugou (Fig. 1) have been further investigated. So far, five families, nine genera and 14 species of Plecoptera have been recorded, including seven new species records to China. In this paper, we also provide a checklist and high-definition colour photos of these stoneflies, with the biology of several common species for the first time, which will be helpful for further biodiversity monitoring and conservation of the local fauna.

## Materials and methods

Specimens were collected by hand, sweep net and light trap and preserved in 75% ethanol. Abdominal segments of specimens were examined and illustrated by KEYENCE VHX-5000. Photographs were taken with a Canon camera (EOS 5D Mark III & PowerShot SX730 HS) and optimised by Adobe Photoshop CS6. The materials are deposited in the Insect Collection of Yangzhou University, Jiangsu Province, China.

Materials examined are from these localities: **Site A1**: Xiaodong Gou Village (Painter′s Village), Benxi County, Liaoning Province, 3-5-VII-2018, 705 m alt., 41°10.589′N, 124°39.438′E, leg. Huo Qing-Bo, Gao Peng. **Site A2**: Xiaodonggou Village, Benxi Autonomous County, Liaoning Province 9-10-VI-2019, 589 m alt., 41°10.806′N, 124°40.148′E, leg. Huo Qing-Bo, Yang Yu-Ben. **Site B**: “Tianguan Virgin Forest”, Benxi County, Liaoning Province, 4-VII-2018, 575 m alt., 41°13.893 ′N, 124°37.353′E, leg. Huo Qing-Bo, Gao Peng. **Site C**: “Daomugou Industrial Area”, Benxi County, Liaoning Province, 5-VII-2018, 705 m alt., 41°15.508′N, 124°43.318′E, leg. Huo Qing-Bo, Gao Peng.

## Checklists

### A checklist of stoneflies from Taizi River

#### Plecoptera

BC9D39AC-0156-5035-B4FD-4AC3C0236FF6

## Analysis

### Taxa

The identification of the stonefly fauna is based on previous taxonomic literature including Teslenko and Zhiltzova (2009), Judson and Nelson (2012), Murányi et al. (2015), Murányi and Hwang (2017). Species names are listed in Table 1, with their distributions revised according to the records by Kim et al. (1998), Teslenko and Zhiltzova (2009), Hwang et al. (2016) and DeWalt et al. (2022). Photos of each species are shown in the figures below.

### Biology of adults

**Emergence and parasite**

The emergence sites are located on riverside plants and structures. Only *Oyamianigribasis* and *Stavsolusmanchuricus* always emerge in the broad mainstream of Taizi River, but other smaller-sized species are more common in the tributaries of width less than 3 m. All the Leuctridae, Nemourinae, Chloroperlidae and *Neoperla* there can be collected on grasses or shrubs (height＜3 m) by the river, while *Oyamianigribasis*, *Stavsolusmanchuricus* and *Isoperlaflavescens* often fly to the higher tree canopy (height＞4 m).

**Figure 14.**
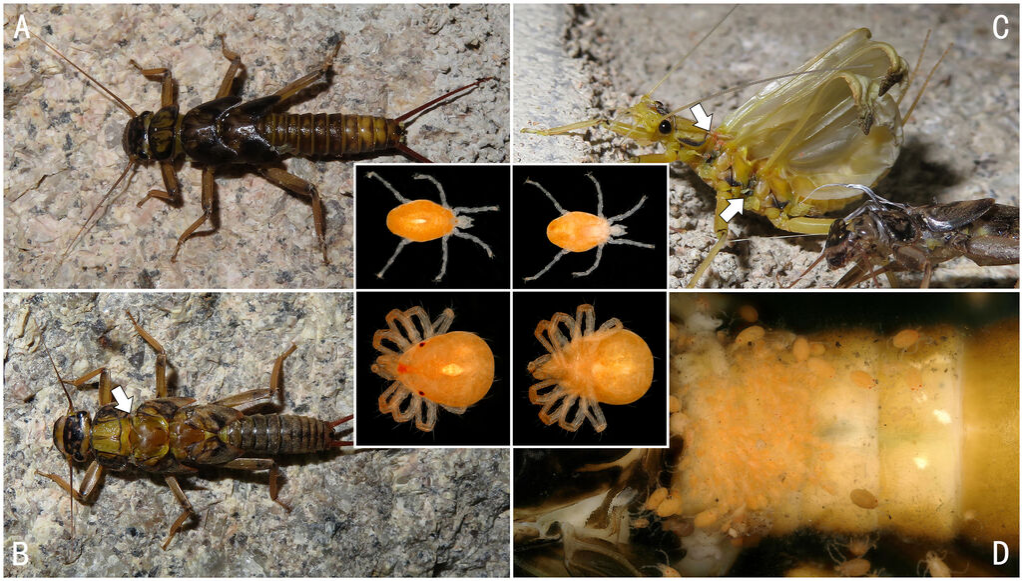
Hydryphantidae sp. on the body of *Oyamianigribasis*. **A** the landing nymph; **B** mites climbing on to the thoracic segment of the emerging stonefly; **C** mites walking to the metathorax and abdomen of the stonefly; **D** multiple mites on terga 1-4 of the stonefly.

In addition, *Oyamianigribasis* is the primary carrier of water mites (Hydryphantidae sp.) and each adult can carry dozens of mites of different instars (Fig. 14). These red mites crawl on stonefly larvae as they emerge. Since the dorsal plate of the thoracic segment appears first during moulting, the mites first reach the junction of the dorsal pronotum and the mesothorax (Fig. 14A and B) and then concentrate on the metathorax and abdominal terga 1 - 3 when the adult wings begin to stretch (Fig. 14C and D). In all the materials, these mites were rarely found in any small-sized stonefly species. We cannot judge whether they have a specific host preference for the time being, but can only speculate that they may prefer to ride on larger and stronger hosts to facilitate the spread of their populations.

### Mating and spawning

**Figure 15.**
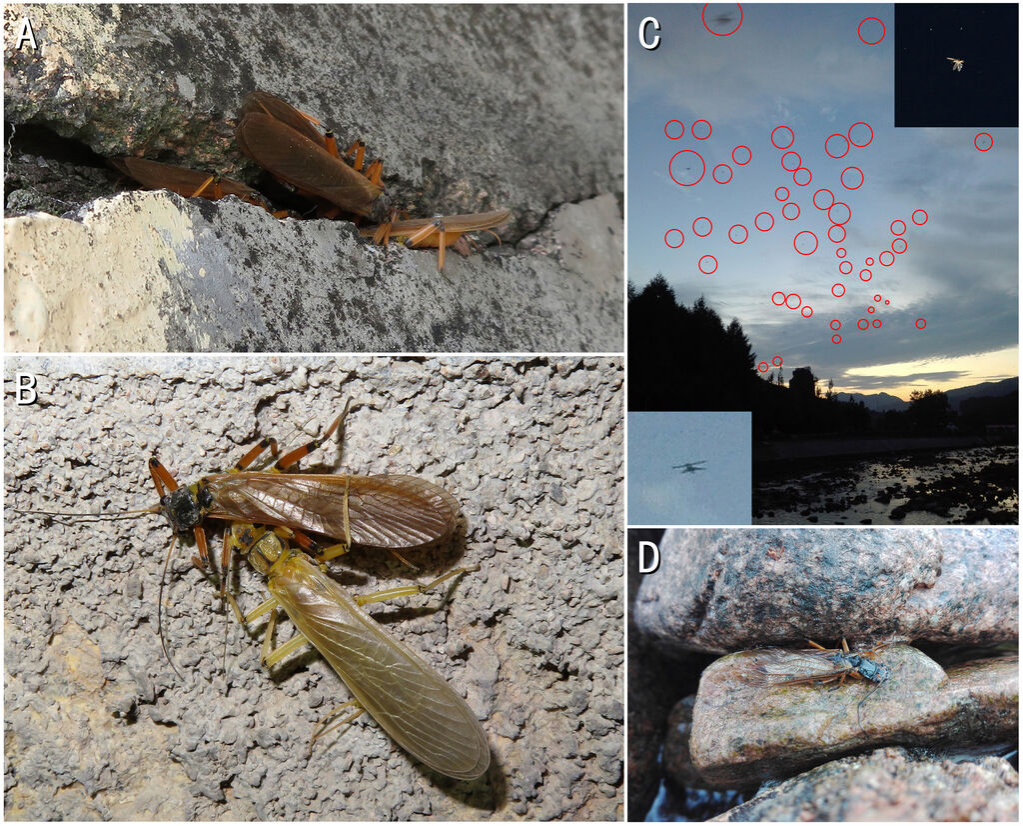
*Oyamianigribasis*. **A** mating ball; **B** a couple of mature male and newly-emerged female; **C** multiple females with eggs in the sky; **D** a dead female after spawning.

Most of the above species are ready to mate after eclosion in early June (the emergence date of *Neoperlacoreensis* is still unknown) and large numbers of females with egg masses can still be observed by early July (Table 2). All species can mate on riverside plants (on leaves or branches), but only *Oyamianigribasis* appeared in groups in crevices of rocks or buildings to compete for mates and usually become the mating ball (Fig. 15A). Mate guarding is also only documented in this species between the mature male and newly-emerged female (Fig. 15B).

In June, females of Chloroperlidae will fly to the river to lay eggs before it becomes dark (19:00 - 19:20 h, Beijing time). In early July, when the sun is setting, but the afterglow can still illuminate the entire river surface (19:30 - 19:50 h, Beijing time), *Oyamianigribasis* females carrying eggs will fly and crash into the river during this time period from the mountains on both sides of the river (Fig. 15C). We used net interception on the bridge and over 50 *Oyamianigribasis* and a few *Stavsolusmanchuricus* were collected. We followed the *Oyamianigribasis* into the Taizi River, but found nothing on the surface; they probably dived to lay their eggs. During the next day, there are often large numbers of dead *Oyamianigribasis* females in the rocks where the river turns, with their abdomen empty (Fig. 15D).

### Phototaxis

**Figure 16.**
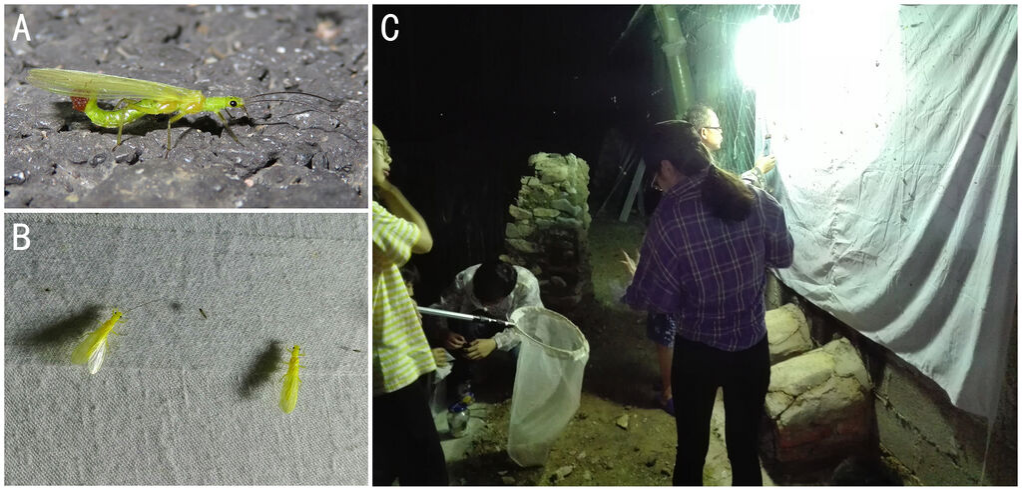
Photographs in the field. **A** *Alloperla* sp. with egg mass; **B** *Suwalliaasiatica* on the curtain; **C** the light trap set in Xiaodonggou.

Amongst the above species, Leuctridae, Nemourinae and *Neoperlacoreensis* were not attracted and collected by light traps. *Oyamianigribasis* and *Stavsolusmanchuricus* have weak phototaxis, even being photonegative to the extent that females carrying eggs were seldom found under the light (n < 5 per night) and immediately changed flight direction or rushed into the river when illuminated by an electric torch. In addition, *Alloperla* spp. and *Suwalliaasiatica* are the most phototactic species there (Fig. 16A–C), either male or female carrying eggs (n > 20 per night), which means that this family may be most vulnerable to human activity along river banks.

### Remarks

Most of the stonefly species mentioned in this article were originally recorded only in the Russian Far East, Korea Island or Mongolia. The Changbai Mountains is on the border with North Korea and close to the Russian border. Therefore, this study presents the potential local diversity of stonefly species and more neighbouring species may be discovered in northeast China in the future.

## Supplementary Material

XML Treatment for Plecoptera

## Figures and Tables

**Figure 2.**
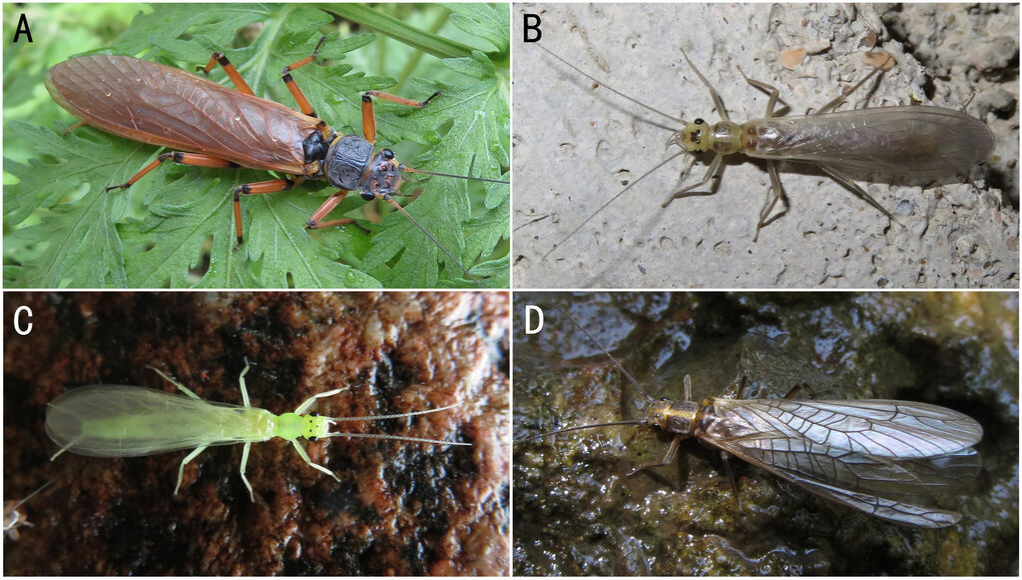
The four most common stoneflies in Xiaodonggou. **A** *Oyamianigribasis* Banks, 1920; **B** *Isoperlaflavescens* Zhiltzova & Potikha, 1986; **C** *Alloperlajoosti* Zwick, 1972; **D** *Stavsolusmanchuricus* Teslenko, 1999.

**Figure 3.**
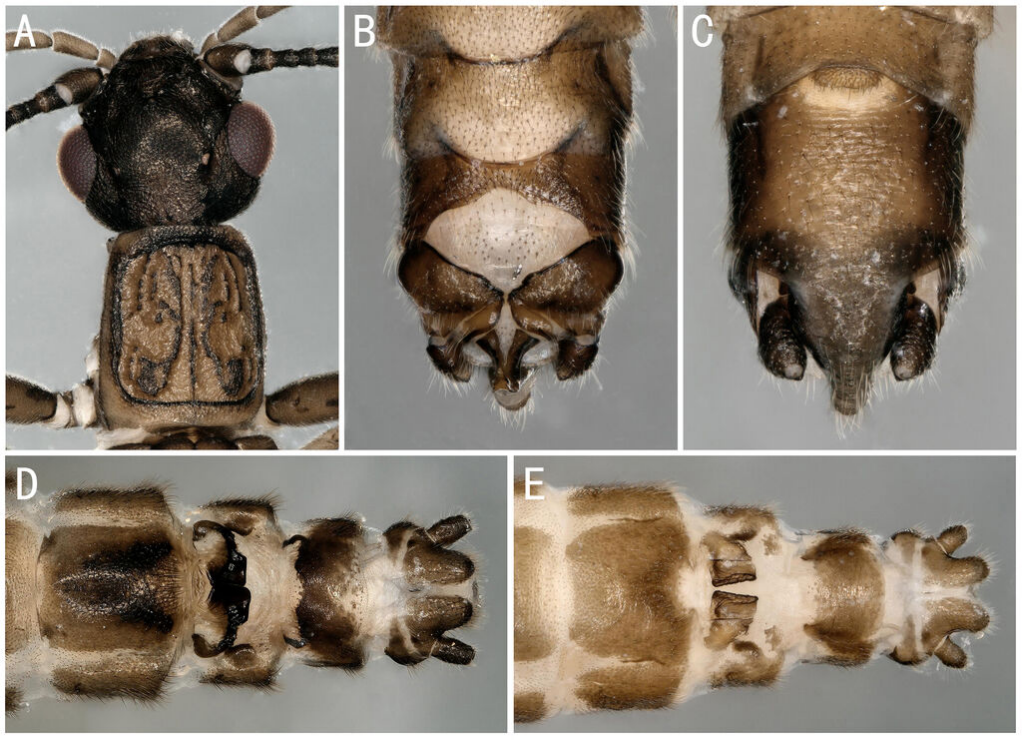
*Perlomyiasmithae* Nelson & Hanson, 1973, *Perlomyiabaei* Murányi & Hwang, 2017 and *Perlomyiakoreana* Murányi & Hwang, 2017. **A–C** *P.smithae*, male head and pronotum; terminalia, dorsal and ventral view; **D** *P.baei*, female terminalia, ventral view; **E** *P.koreana*, female terminalia, ventral view.

**Figure 4.**
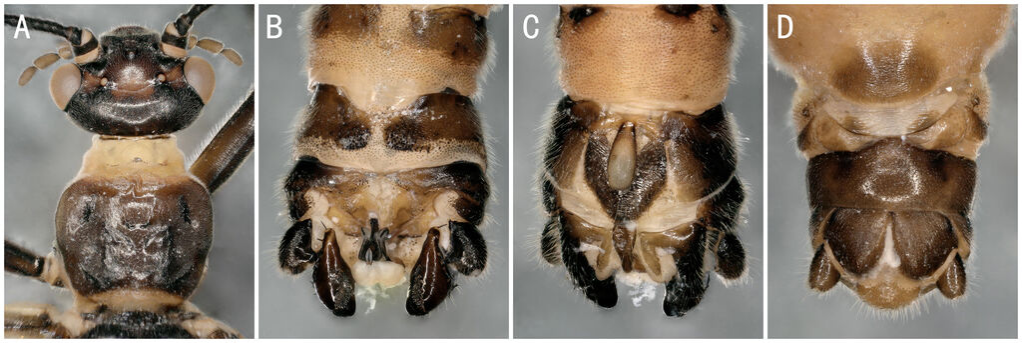
*Amphinemuracoreana* Zwick, 1973. **A** male head and pronotum; **B–C** male terminalia dorsal and ventral view; **D** female terminalia ventral view.

**Figure 5.**
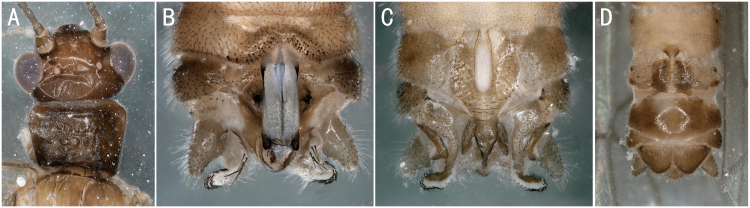
*Amphinemuraverrucosa* Zwick, 1973. **A** male head and pronotum; **B–C** male terminalia dorsal and ventral view; **D** female terminalia ventral view.

**Figure 6.**
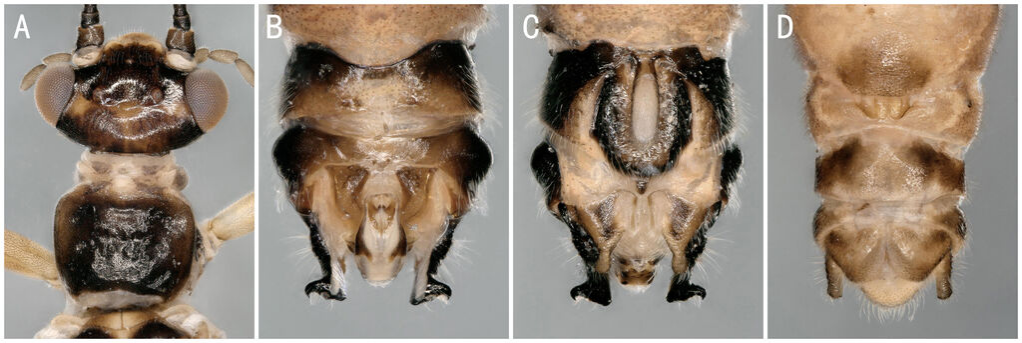
*Nemourapapilla* Okamoto, 1922. **A** male head and pronotum; **B–C** male terminalia, dorsal and ventral view; **D** female terminalia, ventral view.

**Figure 7.**
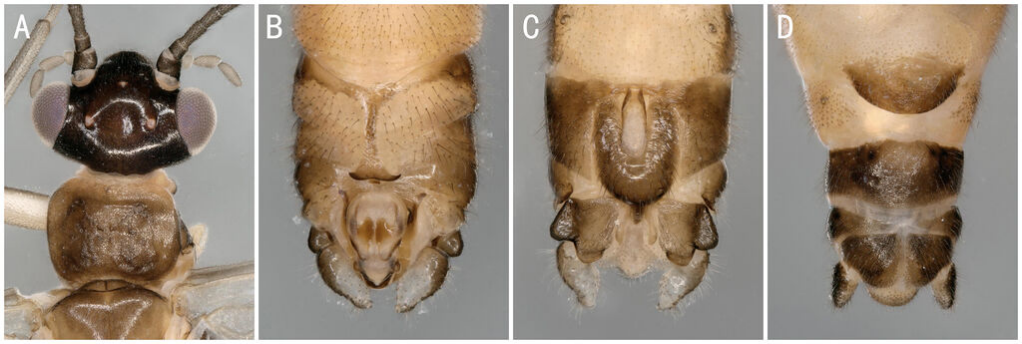
*Nemouratau* Zwick, 1973 **A** male head and pronotum; **B–C** male terminalia, dorsal and ventral view; **D** female terminalia, ventral view.

**Figure 8.**
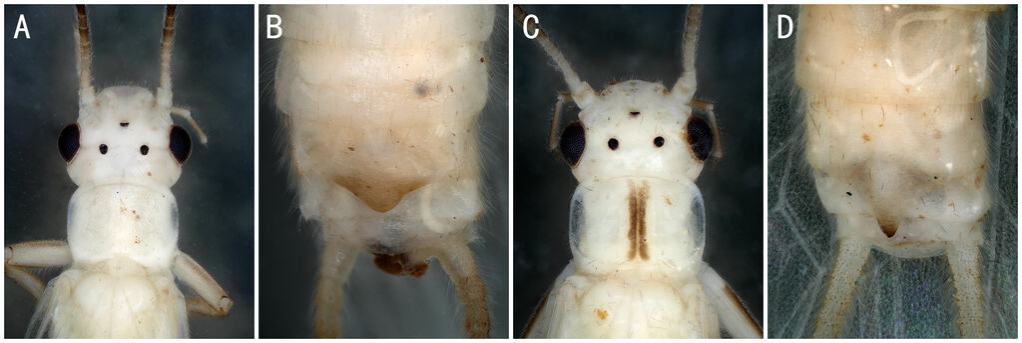
*Alloperlajoosti* Zwick, 1972 and *Alloperlamediata* (Navás, 1925) females. **A–B** *A.joosti* head and pronotum; terminalia ventral view; **C–D** *A.mediata*, head and pronotum; terminalia ventral view.

**Figure 9.**
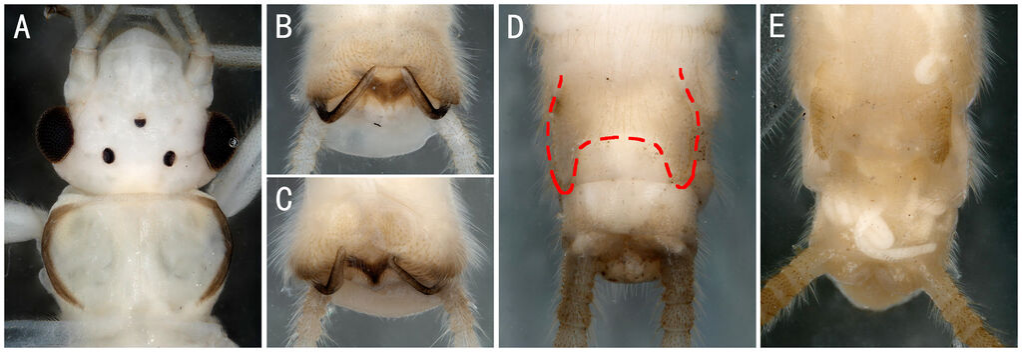
*Suwalliaasiatica* Zhiltzova & Levanidova, 1978. **A** male head and pronotum; **B–C** male terminalia with the shapes of epiproct slightly different dorsal view; **D–E** female terminalia with the subgenital plate slightly/heavily sclerotised ventral view.

**Figure 10.**
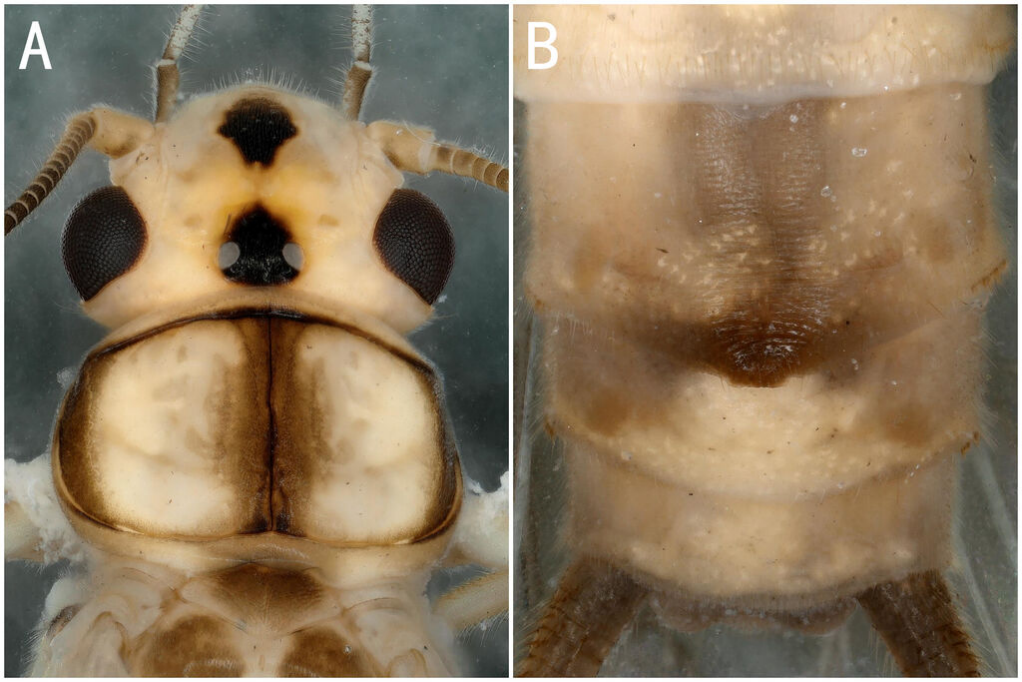
*Neoperlacoreensis* Ra, Kim, Kang & Ham, 1994, female **A** head and pronotum; **B** terminalia ventral view.

**Figure 11.**
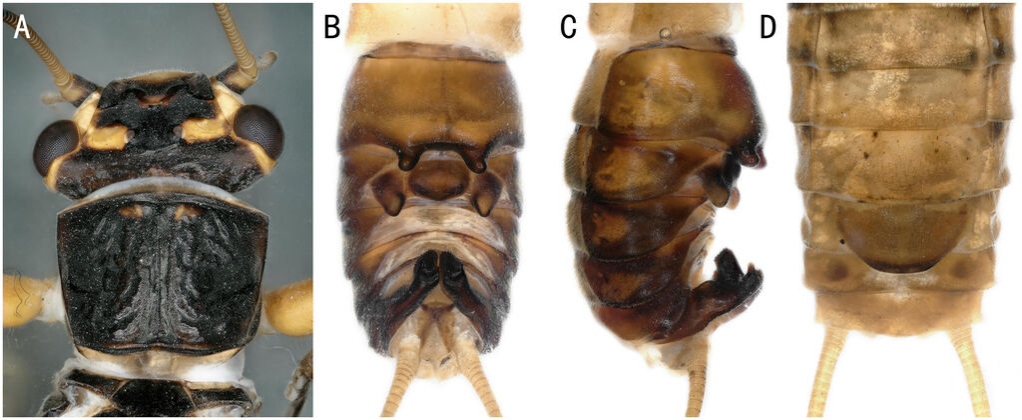
*Oyamianigribasis* Banks, 1920. **A** male head and pronotum; **B–C** male terminalia dorsal and ventral view; **D** female terminalia ventral view.

**Figure 12.**
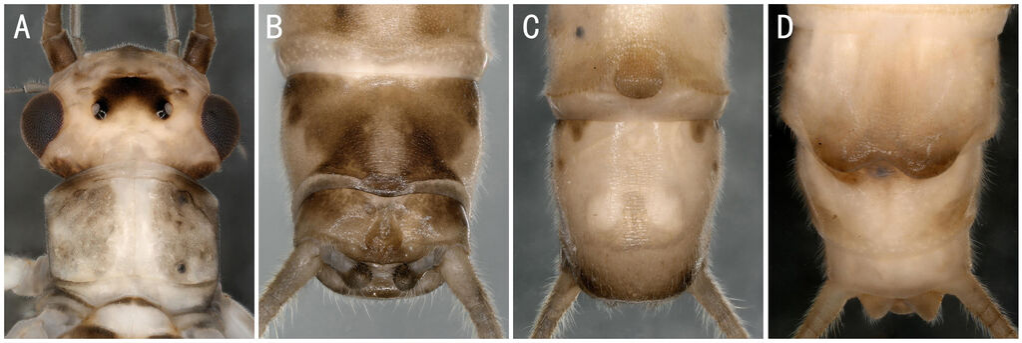
*Isoperlaflavescens* Zhiltzova & Potikha, 1986. **A** male head and pronotum; **B–C** male terminalia dorsal and ventral view; **D** female terminalia ventral view.

**Figure 13.**
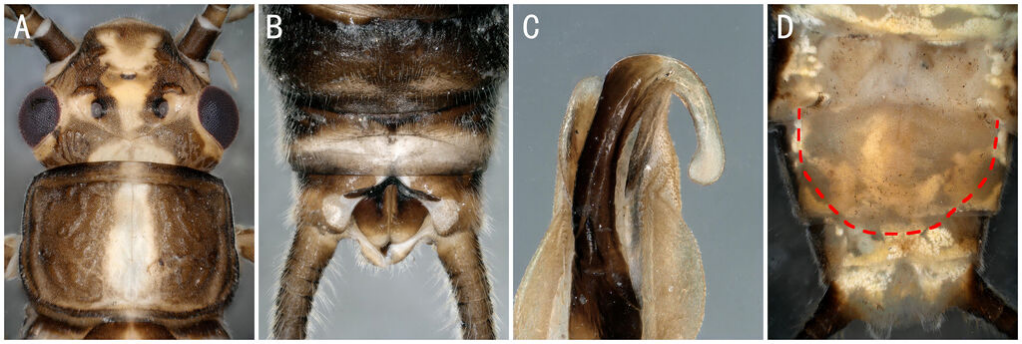
*Stavsolusmanchuricus* Teslenko, 1999. **A** male head and pronotum; **B** male terminalia, dorsal view; **C** apical epiproct; **D** female terminalia, ventral view.

**Table 1.** A checklist of stoneflies from Taizi River.

**Family**	**Species**	**Sites**	**Number**	**Figures**	**Distribution**
**Leuctridae**	*Perlomyiabaei* Murányi & Hwang, 2017	A2	10 females	Fig. 3D	South Korea; China*
	*Perlomyiakoreana* Murányi & Hwang, 2017	A2	2 females	Fig. 3E	South Korea; China*
	*Perlomyiasmithae* Nelson & Hanson, 1973	A2	5 males	Fig. 3A-C	China; North Korea; South Korea; Russia
**Nemourinae**	*Amphinemuracoreana* Zwick, 1973	A2	6 males, 2 females	Fig. 4A-D	South Korea; Kazakhstan; North Korea; Russia; China*
	*Amphinemuraverrucosa* Zwick, 1973	B	2 males, 1 female	Fig. 5A-D	Russia; South Korea; China
	*Nemourapapilla* Okamoto, 1922	A2	3 males, 4 females	Fig. 6A-D	Japan; Russia; South Korea; China
	*Nemouratau* Zwick, 1973	A2	2 males, 2 females	Fig. 7A-D	South Korea; China*
**Chloroperlidae**	*Alloperlajoosti* Zwick, 1972	A1, B	4 females	Fig. 2C, Fig. 8A-B	Russia; Mongolia; South Korea; China*
	*Alloperlamediata* (Navás, 1925)	A1	2 females	Fig. 8C-D	Russia; China; Japan; South Korea; Mongolia
	*Suwalliaasiatica* Zhiltzova & Levanidova, 1978	A1, A2, B	17 males, 20 females	Fig. 9A-D	Russia; China
**Perlidae**	*Neoperlacoreensis* Ra, Kim, Kang & Ham, 1994	A1,	2 females	Fig. 10A-B	South Korea; China*
	*Oyamianigribasis* Banks, 1920	A1, A2, C	25 males, 32 females, 20 nymphs/shed skins	Fig. 2A, Fig. 11A-D	China; Russia; South Korea
**Perlodidae**	*Isoperlaflavescens* Zhiltzova & Potikha, 1986	A1, A2, B	5 males, 3 females	Fig. 2B, Fig. 12A-D	Russia; South Korea; China*
	*Stavsolusmanchuricus* Teslenko, 1999	A1, A2, B	18 males, 20 females	Fig. 2D, Fig. 13A-D	Russia; China; South Korea

**Table 2.** Occurrence of adult stoneflies at Taizi River.

**Taxon/ Month**	Jan	Feb	Mar	Apr	May	Jun	Jul	Aug	Sep	Oct	Nov	Dec
**Leuctridae**												
*Perlomyiamartynovi*	-	-	-	-	-	+	-	-	-	-	-	-
*Perlomyiasecunda*	-	-	-	-	-	+	-	-	-	-	-	-
*Perlomyiasmithae*	-	-	-	-	-	+	-	-	-	-	-	-
**Nemourinae**												
*Amphinemuracoreana*	-	-	-	-	-	+	-	-	-	-	-	-
*Nemourahamulata*	-	-	-	-	-	+	-	-	-	-	-	-
*Nemouratransversospinosa*	-	-	-	-	-	+	-	-	-	-	-	-
*Nemouratau*	-	-	-	-	-	+	-	-	-	-	-	-
**Chloroperlidae**												
*Alloperlaacietata*	-	-	-	-	-	+	-	-	-	-	-	-
*Alloperlakurilensis*	-	-	-	-	-	+	+♀	-	-	-	-	-
*Alloperlamediata*	-	-	-	-	-	+	+♀	-	-	-	-	-
*Suwalliaasiatica*	-	-	-	-	-	+	+	-	-	-	-	-
**Perlidae**												
*Neoperlacoreensis*	-	-	-	-	-	+	+♀	-	-	-	-	-
*Oyamianigribasis*	-	-	-	-	-	+	+	-	-	-	-	-
**Perlodidae**												
*Isoperlaflavescens*	-	-	-	-	-	+	-	-	-	-	-	-
*Stavsolusmanchuricus*	-	-	-	-	-	+	+♀	-	-	-	-	-
